# Novel MOF-based vanadium and 2,2 -bipyridine-4,4 -dicarboxylic acid as phenomenal dye adsorbent and antimicrobial agent

**DOI:** 10.3389/fchem.2025.1524683

**Published:** 2025-01-30

**Authors:** Baraa Mohammed Yaseen, Farag M. A. Altalbawy, Rafid Jihad Albadr, Waam Mohammed Taher, Mariem Alwan, Mahmood Jasem Jawad, Hiba Mushtaq, Khursheed Muzammil, Ahmed Hussein Zwamel

**Affiliations:** ^1^ Department of Medical Laboratory Technics, College of Health and Medical, Technology, Alnoor University, Mosul, Iraq; ^2^ Department of Chemistry, University College of Duba, University of Tabuk, Tabuk, Saudi Arabia; ^3^ National Institute of Laser Enhanced Sciences (NILES), University of Cairo, Giza, Egypt; ^4^ Pharmacy College, Ahl Al Bayt University, Kerbala, Iraq; ^5^ College of Nursing, National University of Science and Technology, Dhi Qar, Iraq; ^6^ Pharmacy College, Al-Farahidi University, Baghdad, Iraq; ^7^ Department of Pharmacy, Al-Zahrawi University College, Karbala, Iraq; ^8^ Pharmacy College, Gilgamesh Ahliya University, Baghdad, Iraq; ^9^ Associate Professor, Department of Public Health, College of Applied Medical Sciences, Khamis Mushait Campus, King Khalid University, Abha, Saudi Arabia; ^10^ Medical Laboratory Technique College, The Islamic University, Najaf, Iraq; ^11^ Medical Laboratory Technique College, The Islamic University of Al Diwaniyah, Al Diwaniyah, Iraq; ^12^ Medical Laboratory Technique College, The Islamic University of Babylon, Babylon, Iraq

**Keywords:** wastewater treatment, Congo red adsorbent, antimicrobial agent, vanadium, metal organic framework

## Abstract

In this study, a new MOF (metal-organic framework) based on vanadium and 2,2-bipyridine-4,4-dicarboxylic acid (V/BP-MOF) was synthesized. Synthesized V/BP-MOF was introduced as a strong adsorbent of Congo Red (CR) and an effective agent in eliminating microbial species. In the investigation of CR absorption activity, several factors such as concentration of V/BP-MOF, pH, time, and temperature were investigated. Antimicrobial evaluations were carried out on Common bacterial strains in wastewater and values of MIC (minimum inhibitory concentration) and MBC (Minimum Bactericidal Concentration) were reported. The V/BP-MOF was confirmed and characterized by EA, EDS, EDS mapping, FT-IR, XRD, TGA, BET, SEM, and TEM. In checking the characteristics of V/BP-MOF, size, specific surface area, and thermal stability were obtained, respectively, 68 nm, 325 m^2^/g, and 320°C. The highest adsorption of CR, at 94%, was obtained at natural pH, ambient temperature, and after 150 min. In kinetic studies, a correlation coefficient of 0.99 was observed with the pseudo-second-order kinetic model, while in isotherm studies, a correlation coefficient of 0.97 was observed with the Freundlich isotherm model. In the biological evaluations, the best inhibition was against *Escherichia coli*, and MIC and MBC were observed as 4 μg/mL and 2 μg/mL, respectively. As a general result, V/BP-MOF can be introduced as a potent absorbent agent of CR dye and antimicrobial properties. Therefore, the compound synthesized in this study can be introduced as a suitable option for the wastewater treatment industry, with multiple capabilities including the removal of chemical pollutants and pathogenic agents.

## 1 Introduction

Congo Red (CR) or sodium salt of 3,3′-[(1,1′-biphenyl)-4,4′-diyl] bis (4-aminonaphthalene-1-sulfonic acid) is an organic compound soluble in water, which is in the azo dye category (Chatterjee et al., 2020). CR was used in the past to dye cellulosic textiles (Ivanovska et al., 2022). CR is known as a biological agent and acid-base indicator, and its most important use can be called diagnostic use (Oladoye et al., 2022). For example, in histology and microscopy, CR dye is used for staining in amyloidosis (Shehabeldin et al., 2023). Another example is that flow cytometry tests can detect Acanthamoeba, Naegleria, and other amoebic cysts (López-Barona et al., 2022). CR is a toxic compound to humans and other living organisms (Siddiqui et al., 2023).

CR is known as a compound that is present in wastewater and does not degrade easily. It is a source of hazardous pollution that threatens human life, other organisms, and the environment (Liu et al., 2022a).

In addition to chemical compounds, other pathogenic agents, such as bacteria, are commonly found in wastewater. Pathogenic bacteria such as *Salmonella*, *Shigella*, *Yersinia enterocolitica*, and *Escherichia coli* are among these bacteria that cause disease in humans and living organisms (Stobnicka-Kupiec et al., 2024). For example, *salmonella* is the cause of one of the most common food poisoning (Bakhshandeh et al., 2022). *Shigella* causes bloody diarrhea (dysentery) (Hmar et al., 2024). *Yersinia enterocolitica* causes enterocolitis (inflammation of the intestine) and ileitis (inflammation of the small intestine) in humans (Fang et al., 2023). *Escherichia coli* is the most common cause of urinary tract infection, accounting for 90% of urinary tract infections in young women (Czajkowski et al., 2021).

Several methods have been reported to remove chemical and pathogenic agents from wastewater and in water treatment (Nasir et al., 2022).

Nanotechnology and nanostructures can be mentioned as one of the new technologies in this field. Various nano compounds such as metal oxide nanoparticles (Naseem and Durrani, 2021), nanotubes (Chahar et al., 2023) and nanofiber (Radoor et al., 2024) have been reported for the removal of hazardous pollutants, dyes, and inhibition of pathogenic bacterial strains from wastewater.

For example, in a recently reported study, CuO nanoparticles, which were synthesized by the green method, were introduced as a strong adsorbent of CR (Jethave et al., 2022).

In another study, silver nanoparticles were synthesized using two fungal species, and their antimicrobial properties against bacterial agents such as *E. coli* were investigated, with promising results reported (Moustafa, 2017).

Activated carbon nanotubes are another nano compound that has been reported to remove hazardous pollutants such as Cr(VI) (Jia et al., 2022).

Metal-Organic Frameworks (MOFs) that are composed of metal and ligands are another category of nano compounds that have been reported for the removal of dyes and the inhibition of pathogenic bacterial strains (Uddin et al., 2021; Hubab and Al-Ghouti, 2024).

In this regard, we can refer to the synthesized Zn-terephthalate MOF that has the property of removing CR (Obayomi et al., 2023).

The Co-MOF with antimicrobial properties against pathogenic bacterial strains has been reported in recent studies (Feng et al., 2023). The MOFs are composed of metal and ligand.

In addition to the wastewater treatment industry and microbial inhibitory properties, other applications of MOFs such as catalysis (Trzebiatowska et al., 2024), sensing (Shafqat et al., 2023), and proton conduction (Ma et al., 2024), have been reported.

Structural physical and chemical characteristics such as high specific surface area and compounds used in the structure of MOFs can be mentioned as important factors in the applications of MOF compounds (Ahmadi et al., 2021; Cai et al., 2021; He et al., 2021).

In general, MOFs are porous compounds with a high specific surface area, and their main structure consists of metal and organic ligands (Chen et al., 2022).

Vanadium is a metal including biological properties such as anticancer activity (Kumar et al., 2024), antioxidant activity (Zhang et al., 2021b), and antibacterial activity (Suma et al., 2020) that have been reported. Also, nanocomposites containing vanadium have been reported as CR absorbers (Makhtar et al., 2024).

Using vanadium and different ligands, the MOF compounds with various applications, such as catalytic properties (Phan et al., 2011) and biological activity (Du et al., 2024), have been synthesized and reported.

Therefore, if we synthesize a MOF using vanadium and a bioactive ligand that has antibacterial properties and can absorb CR, a valuable compound will be synthesized and reported.

In this study, we examined the adsorption properties of CR and the antimicrobial properties of the synthesized MOF against common bacterial strains in wastewater, such as *Salmonella enterica*, *Shigella dysenteriae*, *Y. enterocolitica* and *E. coli*, using vanadium and 2,2-bipyridine-4,4-dicarboxylic acid as a bioactive ligand.

The high specific surface area and the presence of compounds with high absorption properties and high antibacterial properties in the structure of the newly synthesized MOF (Vanadium-2,2-Bipyridine-4,4-dicarboxylic acid-MOF or V/BP-MOF) has given it the ability to have two vital functions in the field of wastewater treatment, such as the absorption of CR and the inhibition of pathogenic bacterial strains such as *Salmonella enterica*, *S. dysenteriae*, *Y. enterocolitica*, and *E. coli*.

## 2 Experimental section

### 2.1 Raw materials and equipment

Vanadium (III) chloride, 2,2-bipyridine-4,4-dicarboxylic acid, CR, antibiotics, and bacterial culture medium were prepared from Sigma/Merck company. The American Type Culture Collection (ATCC) bacterial strains were used in this study.

Elemental analysis, EDS/EDS mapping, FT-IR, XRD, TGA, BET, SEM, and TEM analyses were used to characterization and confirm the structure of the products, which were prepared by LECO TruSpec (Elemental analysis), TESCAN VEGA 3 (EDS/EDS mapping), Thermo AVATAR (FT-IR), Philips PW1730 (XRD), TA Instruments SDT-Q600 (TGA), BEL BELSORP MINI II (BET), TESCAN VEGA 3 (SEM), and Philips CM 120 (TEM), respectively.

The Thermo Biomate 5 UV-Visible spectrophotometer was used to prepare suspensions of bacterial strains and for adsorption studies.

### 2.2 V/BP-MOF (vanadium-2,2-bipyridine-4,4-dicarboxylic acid-MOF) synthesis method

In 20 mL of deionized water, 1 mmol of vanadium (III) chloride and 2 mmol of 2,2-bipyridine-4,4-dicarboxylic acid were stirred at room temperature until the solution became homogeneous. The obtained homogeneous solution was placed in an ultrasonic bath with a power of 300 W for 30 min at room temperature. The obtained novel V/BP-MOF composition was separated by centrifugation and washed three times with a 1:1 mixture of deionized H_2_O and EtOH before being subjected to nanofiltration. It was then dried in an oven at 100°C under vacuum for 4 h (Ahmad et al., 2022; Ramírez-Coronel et al., 2022).

### 2.3 V/BP-MOF dye adsorbent test

To measure the absorption percentage (AP), V/BP-MOF was added to 0.1 L of CR solution in deionized water and stirred. Then, the absorbance was measured at 497 nm using a spectrophotometer, and Equation 1 was applied (Moghaddam-Manesh et al., 2024).

$$AP=\left[\left(C_{1}-C_{2}\right)/C_{1}\right]100$$ (1)

AP = Absorption percentage (%).

C_1_ = Initial CR concentration (mg/L).

C_2_ = Residual CR concentration (mg/L).

### 2.4 V/BP-MOF antimicrobial test

The common pathogenic bacterial strains of wastewater that were examined in this study included *Salmonella enterica* (ATCC 14028), *S. dysenteriae* (ATCC 13313), *Y. enterocolitica* (ATCC 9610) and *E. coli* (ATCC 25922). In the investigations according to CLSI (Clinical and Laboratory Standards Institute), suspension 1 × 10^5^ CFU/mL of the studied strains was prepared in Mueller-Hinton broth at 630 nm, and tests MIC and MBC were performed as follows (Igei et al., 2016; Saadh et al., 2024).

The concentrations of V/BP-MOF prepared and studied in all strains of this study were 1 μg/mL, 2 μg/mL, 4 μg/mL, 8 μg/mL, 16 μg/mL, 32 μg/mL, …, 512 μg/mL suspended in deionized water.

#### 2.4.1 MIC

First, 90 μL Mueller-Hinton broth, 10 μL studied bacterial strain, and 100 μL of V/BP-MOF (each concentration prepared separately in each well) were poured into each well of the microplate (plate 96). It was placed in a shaker incubator at a temperature of 37°C for 36 h. Then, the wells of the microplate were checked. For each studied strain, the lowest concentration at which the contents were clear was reported as the MIC (Afrough et al., 2021; Saadh et al., 2024).

#### 2.4.2 MBC

For each studied strain, the contents of the clear wells of the microplate in the previous step were cultured on Mueller Hinton broth. Then, incubated at 37°C for 72 h. Finally, for each study strain, the concentration at which the study strain did not grow was reported as MBC (Moghaddam-manesh et al., 2021; Saadh et al., 2024).

## 3 Result and discussion

### 3.1 Confirmation and characterization of V/BP-MOF

**FIGURE 1:**
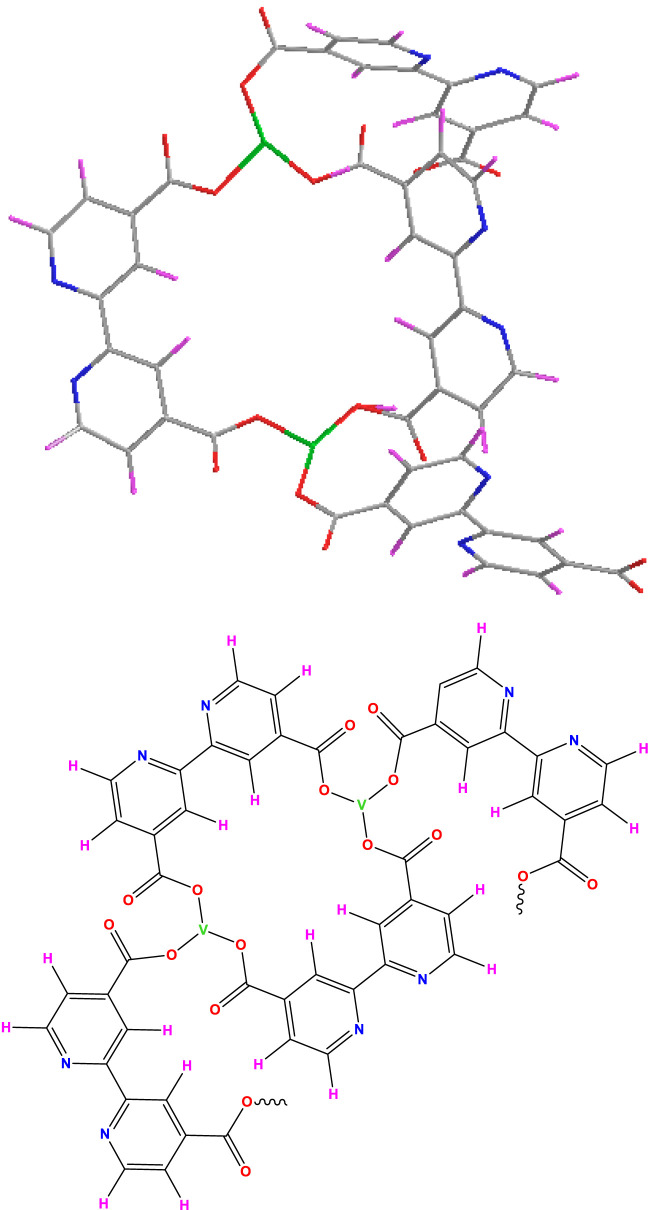
Structure of V/BP-MOF.

For the new V/BP-MOF synthesized in this study, the structure of Figure 1 was proposed. The V/BP-MOF was synthesized from the reaction of vanadium (III) chloride and 2,2-bipyridine-4,4-dicarboxylic acid during the ultrasonic process with a power of 300 W for 30 min at room temperature.

**FIGURE 2:**
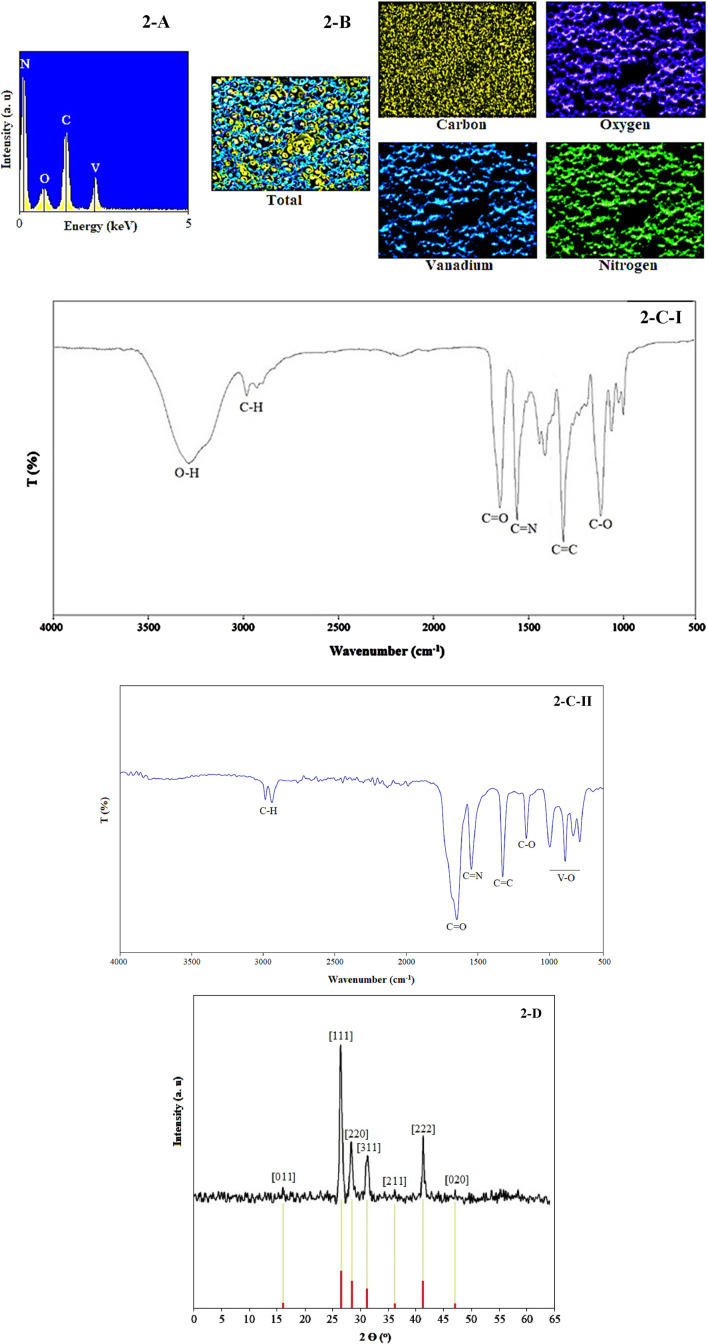
EDS **(A)**, EDS mapping **(B)**, FT-IR **(C)**, and XRD pattern **(D)** of V/BP-MOF.

**FIGURE 3:**
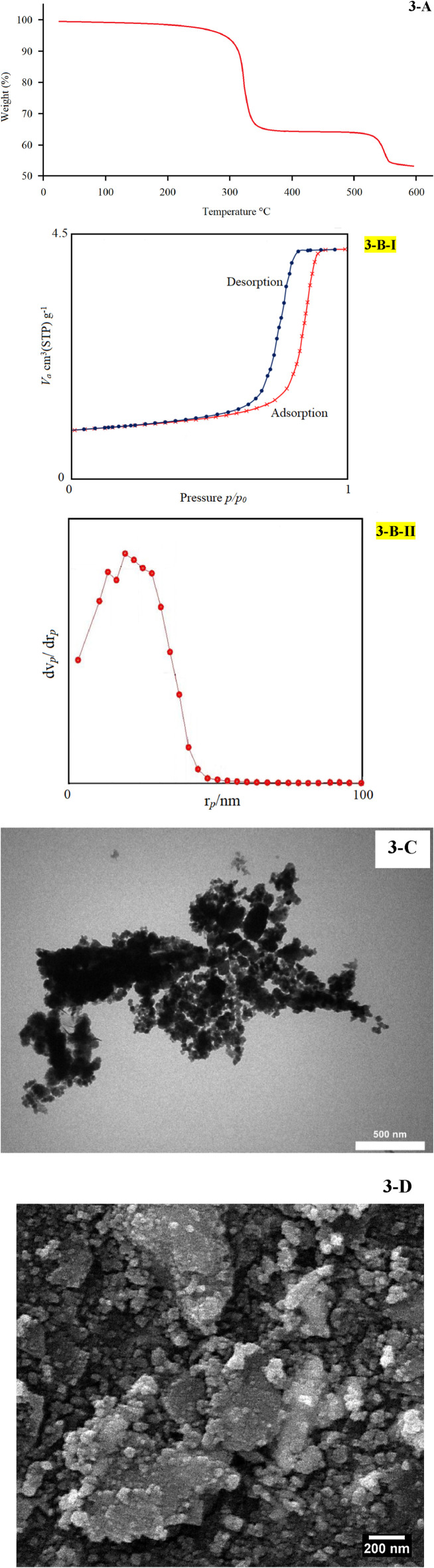
TGA **(A)**, BET **(B)**, TEM **(C)**, and SEM **(D)** of V/BP-MOF.

The predicted structure of Figure 1 and structural features were proved by elemental analysis, EDS (Figure 2A), EDS mapping (Figure 2B), FT-IR (Figure 2C), XRD (Figure 2D), TGA (Figure 3A), BET (Figure 3B), SEM (Figures 3C), and TEM (Figure 3D).

Vanadium-oxygen bonds of the final product were observed in the areas of 650–1,000 cm^−1^ of its FT-IR (Figure 2C–II) spectrum based on previous studies (Chen et al., 2004; Zhang et al., 2015). Referring to previous studies, other links of functional groups such as carbon/hydrogen single bonds, carbon/oxygen doublet bonds, carbon/nitrogen doublet bonds, carbon/carbon doublet bonds, and carbon/oxygen single bonds were observed in nears 3,000–2,950 cm^−1^, 1,625 cm^−1^, 1,520 cm^−1^, 1,385 cm^−1^, and 1,160 cm^−1^ of the FT-IR (Figure 2C–II) spectrum of the V/BP-MOF.

In FT-IR spectrum of 2,2-bipyridine-4,4-dicarboxylic acid (Figure 2C–I) oxygen/hydrogen broad peak, carbon/hydrogen single bonds, carbon/oxygen doublet bonds, carbon/nitrogen doublet bonds, carbon/carbon doublet bonds, and carbon/oxygen single bonds were observed in nears 3,300 cm^−1^, 3,000–2,950 cm^−1^, 1,630 cm^−1^, 1,525 cm^−1^, 1,385 cm^−1^, and 1,150 cm.^−1^

The 2,2-bipyridine-4,4-dicarboxylic acid contains two carboxylic acid groups, which typically exhibit a broad peak (due to the O-H bond) in the region of 3,000–3,500 cm^−1^. The absence of this peak in the FT-IR spectrum of V/BP-MOF suggests that the carboxylic acid groups are bonded through their oxygen of hydroxyl (O-H) groups to vanadium. Furthermore, various bonds such as carbon-hydrogen single bonds, carbon-oxygen double bonds, carbon-nitrogen double bonds, carbon-carbon double bonds, and carbon-oxygen single bonds are present in the structure of 2,2-bipyridine-4,4-dicarboxylic acid. The presence of these bonds in the FT-IR spectrum of V/BP-MOF confirms that this ligand is retained in the final product. Additionally, the presence of vanadium is indicated by a peak associated with vanadium-oxygen bonds in the region below 1,000 cm^−1^.

Based on previous studies, the cubic structure of vanadium (JCPDS card no. 01–076–0,456) was proved in the XRD pattern of the V/BP-MOF (Figure 2D) using peaks 26.7° [011], 26.7° [111], 28.4° [220], 32.6° [311], 37.5° [211], 42.9° [222], and 47.2° [020] in 2theta (Bahlawane and Lenoble, 2014; Li et al., 2020; Kosta et al., 2021; He et al., 2023).

The synthesized V/BP-MOF was stable up to 320°C. The thermal stability of the V/BP-MOF was proved using its TGA curve as shown in Figure 3A. The noticeable weight loss observed in near 320°C, and near 550°C can be attributed to decomposition of 2,2 -bipyridine-4,4 -dicarboxylic acid and destruction of complex network with metal (Saadh et al., 2024), respectively.

According N_2_ adsorption/desorption behavior of sample (Figure 3B–I), the specific surface was obtained as 325 m^2^/g. The N_2_ adsorption-desorption isotherm of sample is similar to type IV according to the IPUAC classification having H_1_ type of hysteresis loop, indicating that the nanostructure has a typical uniform mesopores nature (Thommes et al., 2015).

Based on BJH plot (Figure 3B–II), the porosity behavior of sample was observed in mesopouros area which confirmed results obtained from N_2_ adsorption/desorption of product. (Irwansyah et al., 2024).

In the last technique to determine the structure and characteristics of the V/BP-MOF, its TEM and SEM images were used, as shown in Figures 3C, D, for its morphology and size. The exact morphology and nanosize can be deduced from these images.

Regarding the size of V/BP-MOF, the XRD spectrum and the Debye-Scherer equation were also used, and the size of the final product was calculated to be 68 nm (Al-dolaimy et al., 2024).

As it was proved from the examination of the structural characteristics of the V/BP-MOF by TGA, BET, SEM, and TEM, in this study, a nanostructure with suitable porosity, specific surface area, and suitable thermal stability was synthesized. Previous studies prove these characteristics are induced in the final product based on the synthesis method (Mardkhe et al., 2016; Leng et al., 2021). Therefore, the method used in this study includes ultrasonic treatment at a power of 300 W for 30 min at room temperature, which contributes to these characteristics in the final product and provides evidence of the appropriateness of this method (Ahmad et al., 2022; Ramírez-Coronel et al., 2022).

The porosity and specific surface area, thermal stability and size are important physical and chemical factors in the properties and applications of MOFs (Zhang et al., 2020). Therefore, the applications that have been investigated in the rest of this study on the V/BP-MOF, such as the removal of CR dye and the inhibition of pathogenic bacterial strains in wastewater, can be attributed to the being a nanostructure, having suitable porosity and specific surface area of the synthesized product.

### 3.2 Dye adsorbent activity

The first investigation of the application of the synthesized V/BP-MOF was its application in the absorption of CR.

**FIGURE 4:**
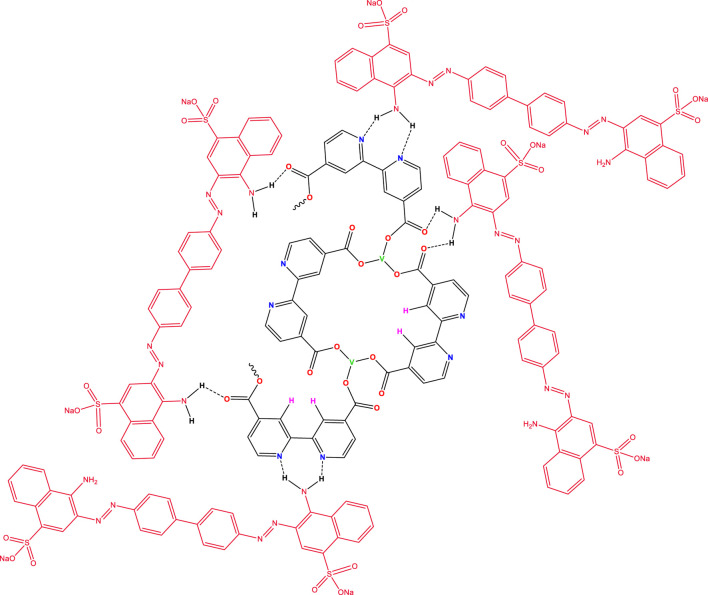
CR’s absorption using V/BP-MOF.

Based on the proposed structure shown in Figure 1 for the V/BP-MOF, the synthetic nanoparticle can lead to the absorption of CR, as shown in Figure 4.

Based on the proposed structure for the absorption of concord, hydrogens attached to the amine groups of CR can form hydrogen bonds with the carbonyl and nitrogen groups of the nanoparticle and lead to its absorption.

To investigate the absorption properties of CR, various tests were performed, and AP (Absorption Percentage) was measured. Experiments and investigations, such as measurements of different CR concentrations, using varying amounts of V/BP-MOF, different pH conditions, different temperatures, and absorption at different times, were carried out.

#### 3.2.1 Investigation of different concentrations of CR

At first, different concentrations of CR in the range of 100 mg/L to 1,000 mg/L were prepared. Under the same conditions, such as the amount of V/BP-MOF, pH, temperature, and time, the absorption of CR was evaluated. For this purpose, the solutions of 100 mg/L, 125 mg/L, 150 mg/L, 300 mg/L, 600 mg/L, and 900 mg/L Conger red were prepared and at ambient temperature (25°C), neutral pH (7), the 0.03 g/L of V/BP-MOF were added and stirred (200 rpm) for 150 min.

**FIGURE 5:**
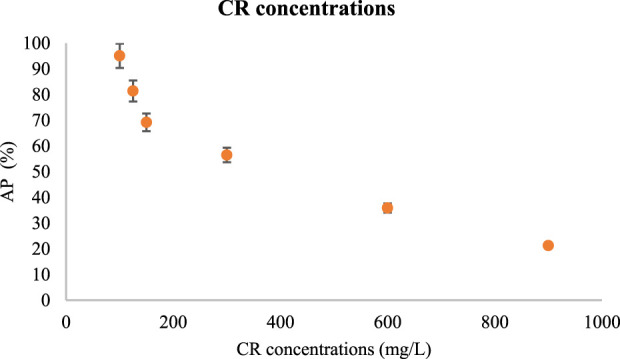
The effect of CR concentration in CR absorption using V/BP-MOF [mean (n = 3) ± SD].

Figure 5 shows the AP values in different concentrations of CR.

Based on the obtained results in concentrations of 100 mg/L, 125 mg/L, 150 mg/L, 300 mg/L, 600 mg/L and 900 mg/L, AP was obtained as 95.1%, 81.4%, 69.2%, 56.5%, 35.9%, and 21.3% respectively.

Therefore, with increasing CR concentration, its absorption decreases. As we know and based on previous studies, nanoparticles have the ability to absorb CR from active sites, which is discussed in detail in Section 3.1. Therefore, with the increase in CR concentration, due to the saturation of the active sites of the V/BP-MOF, its absorption value decreases (Oladoye et al., 2022).

#### 3.2.2 Investigating different amounts of V/BP-MOF in the absorption of CR

In the investigations of the amount of V/BP-MOF, the concentration of CR solution was kept constant at 300 mg/L. Other factors such as temperature (ambient temperature), pH (7), and time (150 min) were also kept constant in all experiments. The amounts of V/BP-MOF was variable and the values of 0.01 g/L, 0.03 g/L, 0.06 g/L, 0.09 g/L and 0.12 g/L were investigated.

**FIGURE 6:**
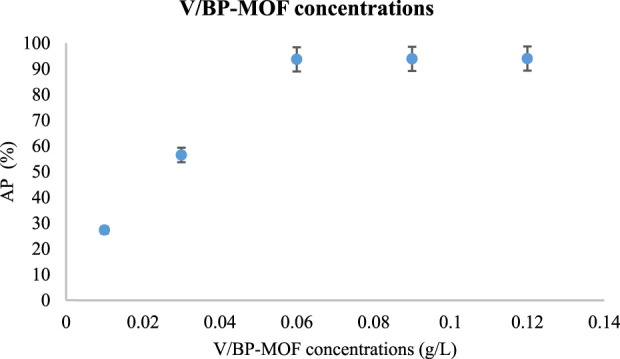
The effect of V/BP-MOF concentration in CR absorption using V/BP-MOF [mean (n = 3) ± SD].

Figure 6 shows the AP values in different amount of V/BP-MOF.

Based on the obtained results at concentrations of 0.01 g/L, 0.03 g/L, 0.06 g/L, 0.09 g/L, and 0.12 g/L of nanoparticles, the absorption percentages (AP) were found to be 27.3%, 56.5%, 93.7%, 93.9%, and 94%, respectively.

These results demonstrate that increasing the amount of V/BP-MOF to 0.06 g/L significantly enhances the absorption capacity. At values above 0.06 g/L, the absorption rate did not show a significant increase and was almost the same.

Therefore, the value of 0.06 g/L (93.7%) can be considered optimal. The lack of high absorption at values higher than 0.06 g/L is due to factors such as the overlap of V/BP-MOF absorption sites and the agglomeration of nanoparticles (Moghaddam-Manesh et al., 2024).

#### 3.2.3 Investigating pH in the absorption of CR

In the tests to investigate the role of pH, the variable was pH. Therefore, the concentration of CR as 300 mg/L, the amount of V/BP-MOF as 0.06 g/L mg/L, the ambient temperature, and the time of 150 min were kept constant in the investigations. The absorption rate of CR was investigated at different pH (4, 5, 6, 7, 9, 8, and 10).

**FIGURE 7:**
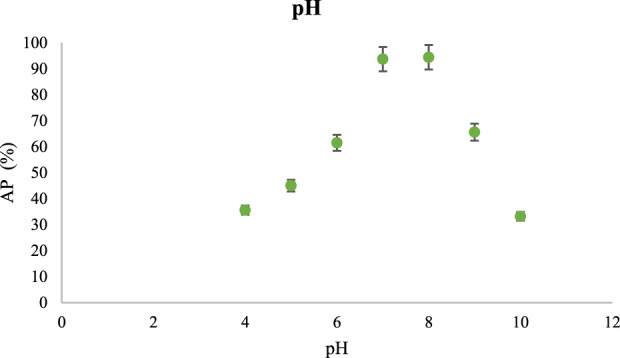
The effect of pH in CR absorption using V/BP-MOF [mean (n = 3) ± SD].

Figure 7 shows the AP values in different pH.

Based on the obtained results in pH of 4, 5, 6, 7, 8, 9, and 10, AP was obtained as 35.6%, 45%, 61.5%, 93.7%, 94.4%, 65.6%, and 33.2% respectively.

As the results indicated, the best absorption occurred at pH 8. In general, the amount of absorption decreases in strong acidic and alkaline pH. Based on the proposed Figure 1, in acidic environment 4, there is a possibility of hydrolysis and breaking of the bond between metal and ligand and destruction of V/BP-MOF (Pessoa and Correia, 2021). However, in other acidic environments, such as 5 and 6, less absorption takes place. The carbonyl groups and nitrogens of the nanoparticles are prone to protonation, which results in reduced absorption of CR (Zhang et al., 2021a). The highest absorption was observed at pH 8. Since the nature of CR is anionic, the negative charge created at this pH can intensify the negative charge of carbonyl oxygen due to the electrophilicity of the carbonyl carbon group and lead to better absorption of CR (Siddique et al., 2024). At alkaline pH 9 and 10, especially at pH 10, since there is a possibility of hydrolysis and breaking of the bond between metal and ligand and destruction of nanoparticle, therefore absorption becomes less (Yesil et al., 2021). So, the lowest absorption was observed at pH 10. In general, since the absorption changes in pH 7 and 8 are not very noticeable, therefore, neutral pH is considered as the optimal condition.

#### 3.2.4 Investigating of temperature in the absorption of CR

Next, the temperature of the absorption process was tested. For this purpose, CR concentration (300 mg/L), amount of V/BP-MOF (0.06 g/L), pH (7), and time (150 min) were kept constant. The absorption process was investigated at ambient temperatures, 30°C, 40°C, 50°C and 60°C.

**FIGURE 8:**
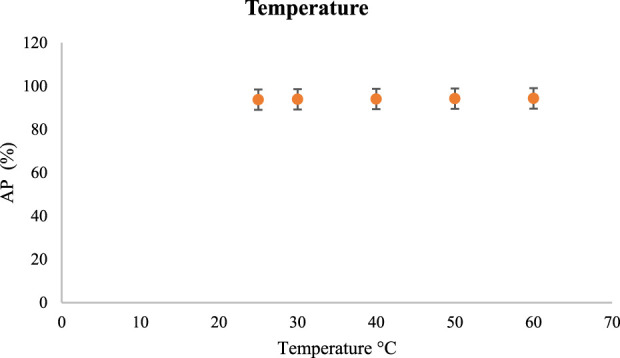
The effect of temperature in CR absorption using V/BP-MOF [mean (n = 3) ± SD].

Figure 8 shows the AP values in different temperatures.

Based on the results of absorption at 25°C 30°C, 40°C, 50°C and 60°C, AP were 93.7%, 93.9%, 94%, 94.2%, and 94.3%, respectively. Therefore, between the ambient temperature and 60°C, the amount of absorption has not increased significantly. Therefore, due to less energy consumption, the ambient temperature was used as optimal.

#### 3.2.5 Investigating process time in the absorption of CR

Finally, the absorption process was evaluated at different times. In these tests, which were performed at 30 min, 45 min, 60 min, 100 min, 150 min, 240 min, and 360 min, CR concentration, V/BP-MOF amount, pH, and temperature were kept constant at 300 mg/L, 0.06 g/L, 7, and 25°C, respectively.

**FIGURE 9:**
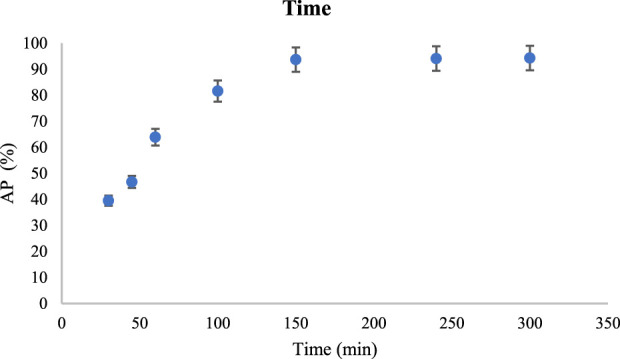
The effect of process time in CR absorption using V/BP-MOF [mean (n = 3) ± SD].

Figure 9 shows the AP values in different time.

Based on the results of absorption at 30 min, 45 min, 60 min, 100 min, 150 min, 240 min, and 360 min, AP were 39.5%, 46.7%, 63.9%, 81.6%, 93.7%, 94.1%, and 94.3%, respectively. By increasing the time to 150 min, the absorption of CR showed a significant improvement. Although it increased slightly up to 360 min, which can be attributed to the remaining sites of the nanoparticle in CR adsorption, 150 min can be reported as the appropriate time for CR adsorption by the nanoparticle.

#### 3.2.6 Adsorption kinetics

In order to investigate the adsorption kinetics, pseudo-first-order (Equation 2), pseudo-second-order (Equation 3), and Elovich models (Equation 4) were used. Their equations are as follows (Musah et al., 2022):

$$\log\left(qe-qt\right)=\log qe-\frac{1}{2.033}K1\;t$$ (2)

qe and qt (mg. g^−1^): The amount of adsorbed at equilibrium and time t.

K1 (g.mg^−1^.min^−1^): The pseudo-first-order rate constant

tqt=1K2 qe2+tqe (3)

K2 (g.mg^−1^.min^−1^): The pseudo-second-order rate constant

qt=a+b⁡ln⁡t (4)

a: *y*-intercept

b: slope of the line.

**FIGURE 10:**
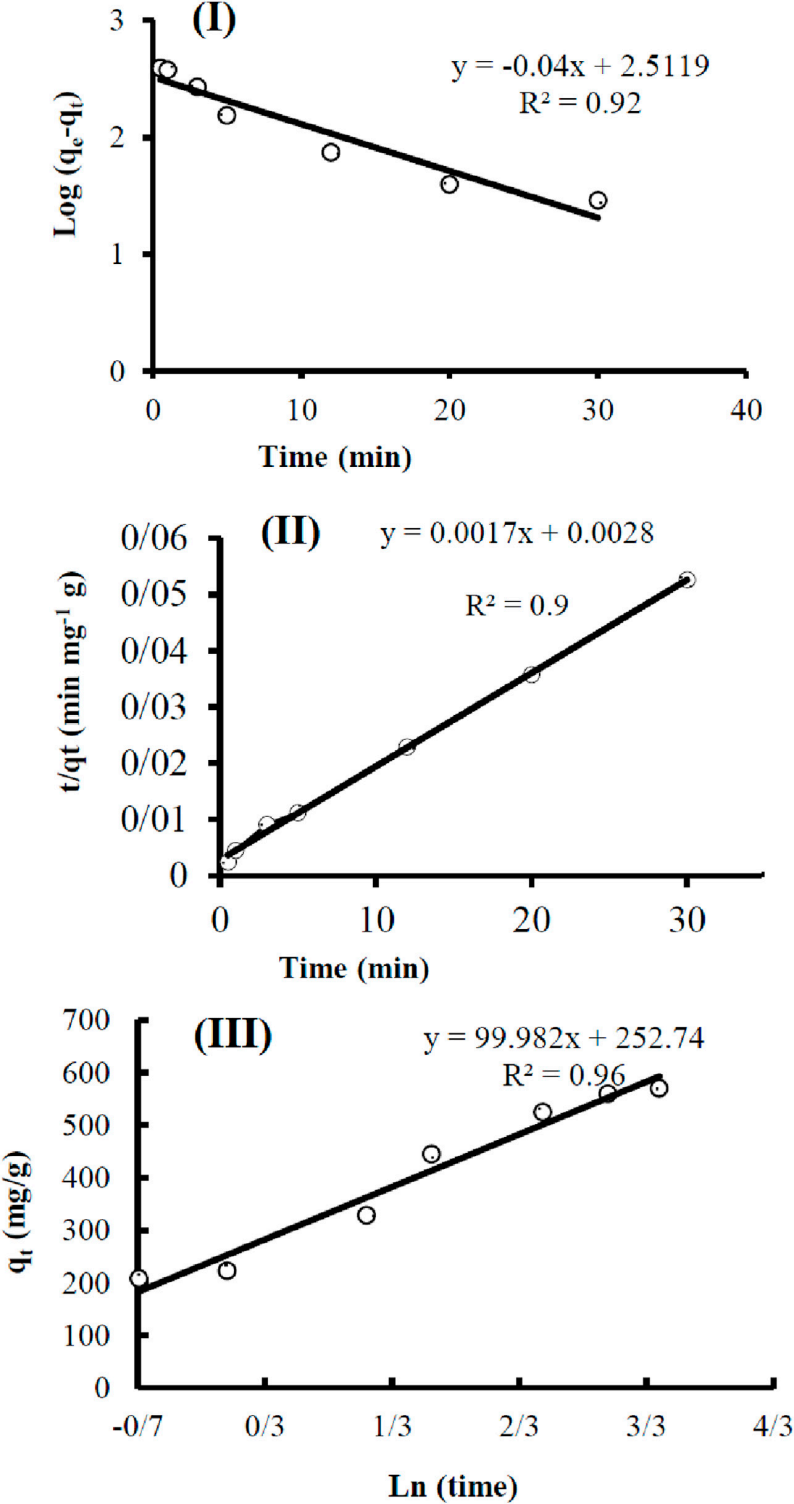
Adsorption kinetic studies: pseudo-first-order **(I)**, pseudo-second-order **(II)**, and Elovich **(III)**.

The results of the pseudo-first-order kinetic model study are presented in Figure 10–I [log(qe-qt) and t]. The results of the pseudo-second-order kinetic model study are presented in Figure 10–II (t/qt and t). The results of the Elovich kinetic model study are presented in Figure 10–III (qt and ln t).

The parameters of the kinetic studies are given in Table 1.

**TABLE 1:** Kinetic studies parameter.

Pseudo-first-order	$\log\left(qe-qt\right)=2.5119-0.04t$	R^2^	qe (mg.mg^−1^)	K1 (g.mg^−1^.min^−1^)
		0.92	325.01	0.092
Pseudo-second-order	tqt=0.0028+0.0017t	R^2^	qe (mg.mg^−1^)	K2 (g.mg^−1^.min^−1^)
		0.99	588.23	0.001
Elovich	$qt=99.982\ln\left(t\right)+252.74$	R^2^	A	b
		0.96	9.e9	0.16

Based on the obtained data, the pseudo-second-order kinetic model fits the data better, as indicated by a correlation coefficient of 0.99.

Therefore, adsorption occurs nonlinearly and at a high rate, significantly influenced by the concentration of the adsorbate. This model is commonly used to describe adsorption processes on solid surfaces and is applicable in the field of water purification (Thottathil et al., 2024).

#### 3.2.7 Adsorption isotherms

In order to investigate the adsorption isotherms, Langmuir (Equation 5), Freundlich (Equation 6), and Temkin (Equation 7) were used. Their equations are as follows (Al-Ghouti and Da’ana, 2020):

$$\frac{Ce}{qe}=\frac{1}{KL\;qmax}+\frac{1}{qmax}Ce$$ (5)

qe (mg. g^−1^): The amount of adsorbed at equilibrium.

Ce (mg. g^−1^): The equilibrium concentration.

KL: Langmuir adsorption equilibrium constant

$$\log\text{ qe}=\log KF+\frac{1}{n}\log Ce$$ (6)

KF: Freundlich adsorption equilibrium constant

n = exponent of the adsorption intensity

qe=B1 ln KT+B1 lnCe (7)

KT: Temkin adsorption equilibrium constant.

B1 = It is calculated from RT. b1^−1^ and b1 is the adsorption temperature.

**FIGURE 11:**
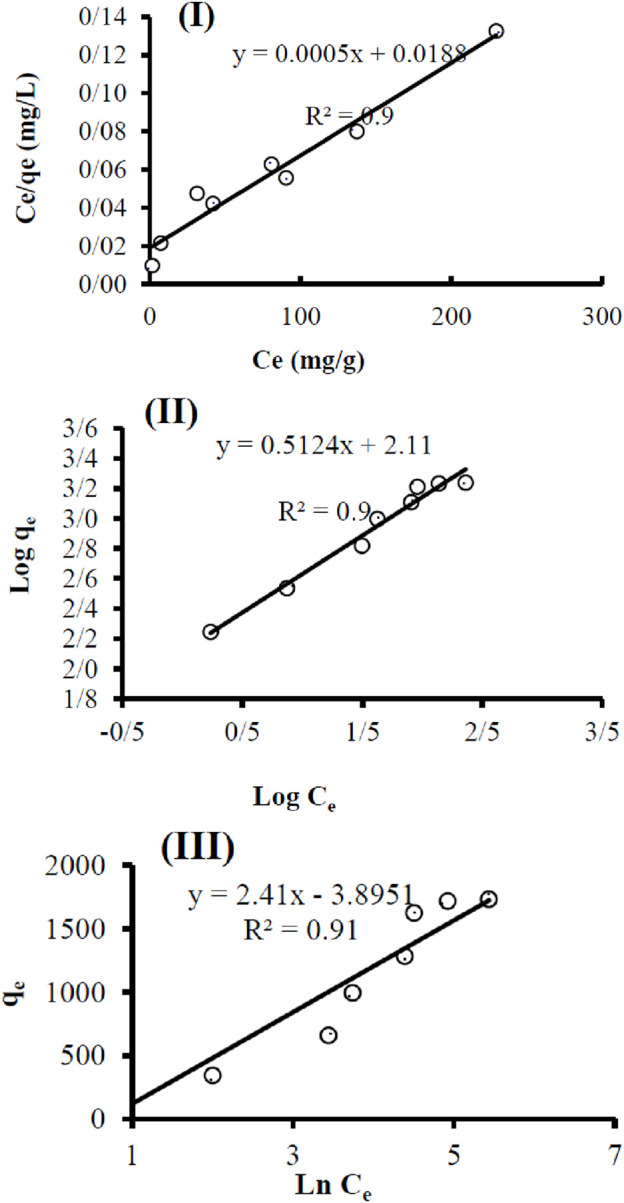
Adsorption isotherm studies: Langmuir **(I)**, Freundlich **(II)**, and Temkin **(III)**.

The results of the Langmuir isotherm study are presented in Figure 11–I Ce/qt and Ce). The results of the Freundlich isotherm study are presented in Figure 11–II (log qe and log Ce). The results of the Temkin isotherm study are presented in Figure 11–III (qe and ln Ce).

The parameters of the isotherm studies are given in Table 2.

**TABLE 2:** Isotherm studies parameter.

Langmuir	$\frac{Ce}{qe}=0.0005Ce+0.0188$	R^2^	Qm (mg.g^−1^)	KL (L.mg^−1^)
		0.96	2,000	0.026
Freundlich	log⁡qe=2.11+0.5124⁡log⁡Ce	R^2^	1/n	KF (L.mg^−1^)
		0.97	0.5124	128.82
Temkin	qe=2.41⁡ln⁡Ce−3.8951	R^2^	B1	KT
		0.90	2.41	4.97

Based on the obtained data, the Freundlich isotherm model fits the data better, as indicated by a correlation coefficient of 0.97.

The Freundlich isotherm model is suitable for describing adsorption processes on heterogeneous surfaces, where different types of adsorption sites are present. This model is effective for low to moderate concentrations of adsorbed substances. This model is applicable in the field of water purification (Al-Ghouti and Da’ana, 2020).

#### 3.2.8 Comparison of CR absorption of V/BP-MOF with some compounds

The highest AP of 0.06 g/L nanoparticles synthesized in this study under optimum conditions was 281.1 mg/L of 300 mg/L of CR solution, which can be said to be nearly 94% (93.7%) absorption. Therefore, the ratio of initial dye concentration to adsorbent dosage is equal to 1,405.5 mg/mg which is a significant amount. Table 3 shows the comparison of the ratio of initial CR concentration to V/BP-MOF with the ratio of initial CR concentration to adsorbent dosage due to some compounds that have been reported recently.

**TABLE 3:** Antibacterial activity of V/BP-MOF against some wastewater strains.

Reported adsorbent composition	Reported absorption capacity (mg/g)
FHGEL	715
Kaolinite supported CoFe_2_O_4_ nanoparticles	390
Nano MnO_2_ in carbon microspheres	308
V/BP-MOF	1,405.5

Therefore, it can be concluded that the synthesized V/BP-MOF has higher absorption property and better performance than some of the recently reported methods.

This high properties of V/BP-MOF in the absorption of CR can be attributed to some physical and chemical characteristics of the synthesized substance, such as its high specific surface area and its constituent compounds (Li et al., 2022).

The high specific surface area allows the V/BP-MOF to be in contact with more CR molecules and leads to more of their absorption (Duojie et al., 2024).

Another factor in this property can be the functional groups in the structure of the V/BP-MOF that cause hydrogen bonding with CR (Liu et al., 2022b).

## 4 Antimicrobial activity

**FIGURE 12:**
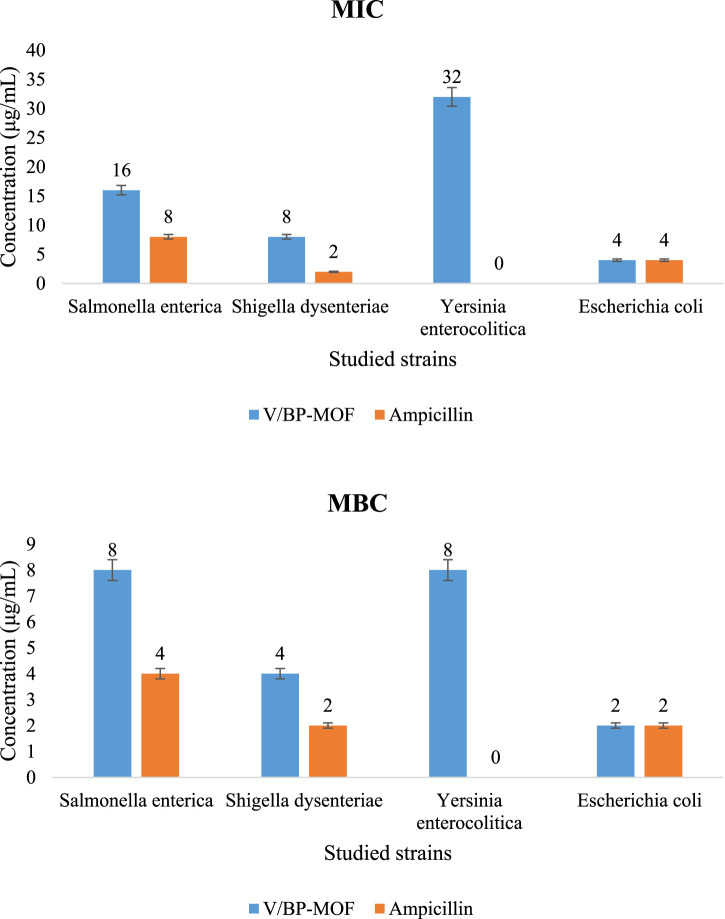
Antibacterial activity of V/BP-MOF against pathogenic bacterial strains in wastewater [mean (n = 3) ± SD].

The inhibition of *Salmonella enterica*, *S. dysenteriae*, *Y. enterocolitica*, and *E. coli*, which are considered significant pathogenic bacterial strains in wastewater, was investigated by the synthesized V/BP-MOF. The MIC and the MBC were examined. The results are shown in Figure 12. Investigations were carried out on concentrations of 1 μg/mL to 512 μg/mL of V/BP-MOF.

The MIC and MBC values of V/BP-MOF against *Salmonella enterica*, *S. dysenteriae*, *Y. enterocolitica*, and *E. coli* were observed as 16 μg/mL and 8 μg/mL, 8 μg/mL and 4 μg/mL, 32 μg/mL and 8 μg/mL, 4 μg/mL, and 2 μg/mL, respectively.

Ampicillin, as a common antibiotic, was used to compare its effectiveness with that of synthesized V/BP-MOF. The result proved that ampicillin is ineffective against *Y. enterocolitica*, but the V/BP-MOF showed good effectiveness.

Part of this acceptable antibiotic activity of V/BP-MOF, as discussed in Section 3, can be attributed to its structural features, such as porosity and specific surface area. As mentioned in previous studies, by increasing the porosity and specific surface area, the contact surface of the nanoparticle with bacterial strains increases and leads to an increase in its inhibitory properties (Staroń and Długosz, 2021; Zheng et al., 2021).

Another significant part of the high antimicrobial property of the synthesized nanoparticle can be attributed to the presence of vanadium and 2,2-bipyridine-4,4-dicarboxylic acid in the final product. According to studies and reports, vanadium and 2,2-bipyridine-4,4-dicarboxylic acid and its compounds have strong antimicrobial properties (Domyati et al., 2021; Mahadevi et al., 2022; Efunnuga et al., 2024; Saadh et al., 2024).

## 5 Conclusion

In the present study, a new MOF containing vanadium and 2,2-bipyridine-4,4-dicarboxylic acid was synthesized (V/BP-MOF). The investigation of its structure via elemental analysis (EA), EDS, EDS mapping, FT-IR, XRD, TGA, BET, TEM, and SEM was confirmed; high thermal stability, high porosity, a large specific surface area, and a well-defined nanostructure were observed. The high absorption capability of Congo Red (CR) was the first application observed from the synthesized V/BP-MOF (94%). Factors such as pH, temperature, and time were analyzed in the absorption studies. Finally, it was proved that the best absorption occurs at ambient temperature, natural pH, for 150 min. The presence of hydrogen bonding sites in the final product, as well as the high porosity and specific surface area, was cited as the reason for the high adsorption properties of CR by the V/BP-MOF. Based on adsorption kinetics and adsorption isotherms studies, pseudo-second-order kinetic and Freundlich isotherm model were proposed for the adsorption process. Microbiology evaluations were carried out on pathogenic bacterial strains of wastewater such as *Salmonella enterica*, *S. dysenteriae*, *Y. enterocolitica*, and *E. coli* in MIC and MBC criteria. The obtained results showed that the MIC for *Salmonella enterica, S. dysenteriae, Y. enterocolitica, and E. coli* were 16 μg/mL, 8 μg/mL, 32 μg/mL, and 4 μg/mL, respectively, indicating the high antimicrobial properties of the synthesized compound. Factors such as bioactive compounds in the structure of the final product, porosity, high specific surface area, and nanoscale size which increases contact with bacteria can be cited as reasons for the high biological activity of the V/BP-MOF. The novelty of this work can be attributed to the report of a new combination with multiple unique capabilities in wastewater treatment and the clean environment goals. In the continuation of the research, it can be suggested to investigate the absorption of other dangerous chemical compounds and bacterial pathogens using synthetic nanoparticles in this study.

## Data Availability

The original contributions presented in the study are included in the article/supplementary material, further inquiries can be directed to the corresponding author.
